# Post-Isometric Back Squat Performance Enhancement of Squat and Countermovement Jump

**DOI:** 10.3390/ijerph191912720

**Published:** 2022-10-05

**Authors:** Michał Spieszny, Robert Trybulski, Piotr Biel, Adam Zając, Michał Krzysztofik

**Affiliations:** 1 Institute of Sports Sciences, University of Physical Education in Krakow, 31-571 Krakow, Poland; 2 Provita Zory Medical Center, 44-240 Zory, Poland; 3 Department of Medical Sciences, The Wojciech Korfanty School of Economics, 40-659 Katowice, Poland; 4 Department of Sport and Physical Education, AGH University of Science and Technology, 30-059 Krakow, Poland; 5 Institute of Sport Sciences, The Jerzy Kukuczka Academy of Physical Education in Katowice, 40-065 Katowice, Poland; 6 Faculty of Physical Education and Sport, Charles University, 162 52 Prague, Czech Republic

**Keywords:** PAPE, post-activation potentiation, countermovement jump, squat jump, complex training

## Abstract

The effectiveness of isometric conditioning activity (CA) is not well described in terms of the level of performance enhancement and the presence of a stretch and shortening cycle in subsequent explosive tasks. Therefore, the aim of this study was to evaluate the effect of a maximum isometric squat as the CA and a subsequent squat jump (SJ) and countermovement jump (CMJ) height. A total of 31 semi-professional handball and soccer players were randomly assigned to two different conditions: (i) 3 sets of 3 repetitions (each lasting 3 s) of maximum isometric back squats (EXP), and (ii) no CA (CTRL). The jump height measurements were performed 5 min before the CA and approximately at the 4th and 8th minute following the completion of the CA. Due to the high inter-individual variability in the potentiation responses, the best value obtained post-CA was also analyzed. The SJ height significantly increased from baseline to the 8th minute post-CA (*p* = 0.004; ES = 0.31; Δ = +3.1 ± 5.0%) in the EXP condition. On the other hand, the CMJ height was significantly higher in the 4th (*p* = 0.001; ES = 0.23; Δ = +2.7 ± 3.7%) and 8th minute post-CA (*p* = 0.005; ES = 0.32; Δ = +3.6 ± 5.7%) in comparison to baseline during the EXP condition. Furthermore, SJ height significantly increased from baseline to the best time-point during the EXP (*p* < 0.001; ES = 0.47; Δ = +4.9 ± 4.9%) and CTRL (*p* = 0.038; ES = 0.21; Δ = +2.5 ± 5.8%) condition. Moreover, the CMJ height was significantly higher at the best time-points than at the baseline during EXP (*p* < 0.001; ES = 0.53; Δ = +5.6 ± 4.7%) and CTRL (*p* = 0.002; ES = 0.38; Δ = +3.1 ± 5.2%) condition. The findings from this study indicate that a maximum isometric squat, used as a CA, effectively improved SJ and CMJ height. This suggests that the presence or absence of a stretch and shortening cycle in both CA and post-CA tasks does not significantly impact the post-activation performance enhancement response.

## 1. Introduction

An effective training method to improve explosive performance is to employ conditioning activity (CA) before a similar movement task, for instance, high-loaded back squats performed before vertical jumping [1]. This performance gain is typically referred to as a post-activation performance enhancement (PAPE). One of the most frequently indicated factors determining the effective induction of the PAPE effect is the principle of similarity between the exercises used [1,2,3]. The rationale is that this effect is mainly local [4] and may be explained by changes occurring in the exercising muscles, such as increased temperature and fluid shifts, which modify muscle metabolism and contraction mechanics [5]. Therefore, the Cas used as a part of a pre-competition or pre-training warm-up should involve the same muscle groups that will be involved in the subsequent training task. Recent studies indicate that the requirements for exercise similarity may be greater than it seems, and such variables as a range of motion [6,7,8], force vector [9,10,11], muscle contraction type, or utilization of the stretch and shortening cycle (SSC) [12,13,14,15] may have a significant impact on the magnitude of the PAPE effect. In addition, the PAPE response is determined by the volume and intensity of the CA [1] and warm-up [16], the rest interval within the complex [17], as well as the individual characteristics of the athlete (i.e., strength level) [18].

Although the impact of PAPE has been thoroughly examined, most of the analysis examined post-CA exercises involving or not involving the SSC, but to the best of the authors’ knowledge, only one study directly compared these conditions [18]. For instance, Rixon et al. [19] found enhanced countermovement jump (CMJ—with SSC) performance following maximum isometric squats as a CA (3 sets of 3 s) in males and females with and without resistance training experience. In turn, after the same CA, Arabatzi et al. [20] indicated increased squat jump (SJ—without SSC) performance among males, with no effects in females, preadolescents, and adolescents of both sexes. In contrast, Chiu et al. [18] compared the acute effect of high-loaded back squats (5 sets of single repetition at 90% one-repetition maximum) on SJ and CMJ among athletic and recreationally trained participants. No effect for an SJ with a decrease in performance during CMJ was noted when all participants were analyzed together. However, the separate analysis of athletes showed that both types of activities were similarly potentiated, with a slightly greater effect during an SJ among athletes. On the other hand, a recent study by Nishioki and Okada [21] reported increased CMJ but no enhancement in SJ performance after 5 sets of 4 repetitions of jump squats (with SSC utilization) at 40% one-repetition maximum as a CA. The authors suggested that it might be attributed to the utilization of SSC during CA and CMJ, but a lack of it during an SJ. They found that CA improved the rate of force development, velocity, and power during the eccentric phase of the CMJ, resulting in enhanced SSC utilization. Since that muscle power generated during the concentric phase highly depends on the effective use of the SSC [22], this explains improved CMJ but not SJ performance. However, it has to be mentioned that Nishioka and Okada [21] investigated the delayed potentiation effect; thus, the performance was tested 24 h post-CA, not within minutes after CA, like in a traditional PAPE protocol. Nevertheless, it cannot be ruled out that such a scenario would also occur during the PAPE response. As evidence from studies on the effects of PAPE on movements performed with and without SSC is sparse and somewhat contradictory, it remains unclear whether one of these activities might gain more from inducing PAPE than the other. 

Other perhaps important aspects regarding the PAPE effect are range of motion and muscle contraction type. Considering the former, Krzysztofik et al. [6] found that when the same range of motion was used in the CA (bench press) as in the subsequent task (bench press throw), a greater PAPE effect was obtained compared to exercises with a limited or extended range of motion. In turn, Esformes and Bampouras [7] reported improvements in CMJ performance after the quarter and parallel squat exercise as the CA. However, the parallel back squat was superior to the quarter back squat, although the preferred squat depth during vertical jumping is more similar to the quarter squat. In regards to the influence of contraction type, Ulrich and Parstorfer [23] compared the effects of CAs (3 repetitions of the bench press) with eccentric-concentric (80% one-repetition maximum) and eccentric-only contractions (120% one-repetition maximum) on power output during the bench press throw. Compared to baseline, power output was significantly enhanced only after the CA with the eccentric-concentric contractions, thus similar to the post-CA. On the other hand, Bogdanis et al. [15] showed that only the isometric CA was effective in the enhancement of subsequent CMJ performance compared with concentric-only and eccentric-only exercise modes. Additionally, Suchomel et al. [14] indicated that ballistic concentric-only half squat potentiated SJ performance to a greater extent than a half squat performed in a non-ballistic manner.

Considering the inconsistent findings, there is a need to clarify whether the presence of SSC in post-CA activity may distinguish the PAPE response. Therefore, the aim of this study was to evaluate the effect of a maximum isometric squat as the CA and a subsequent SJ (without SSC) with a fixed knee angle (same as during the CA) and a non-restricted CMJ (with SSC). It was hypothesized that both jump heights would be enhanced, but a greater effect would be observed in the case of the squat jump.

## 2. Materials and Methods

### 2.1. Experimental Approach to the Problem

**Figure 1:**
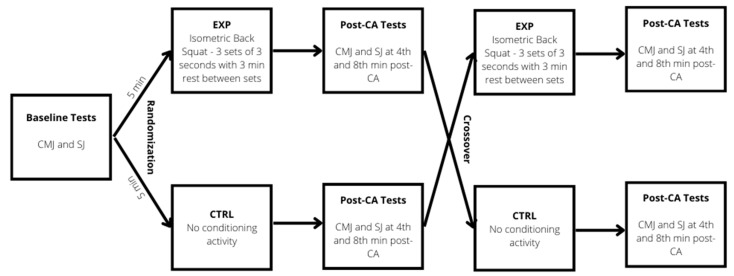
Study design flowchart. CMJ—countermovement jump; SJ—squat jump; EXP—experimental condition; CTRL—control condition; CA—conditioning activity.

The study was performed following a randomized crossover design, where each participant performed two experimental sessions to compare the acute effects of maximum isometric back squat as the CA on post-activation CMJ and SJ height. Participants were randomly assigned to two different conditions: (i) 3 sets of 3 repetitions (each lasting 3 s) of maximum isometric back squats (EXP), and (ii) no CA (CTRL). Measurements were performed 5 min before the CA and approximately at the 4th and 8th minute following the completion of the CA. In the CTRL, measurements were performed at the same time point, but no CA was applied (Figure 1). 

### 2.2. Participants

A total of 31 male semi-professional handball and soccer players participated in this study (Table 1). The inclusion criteria were as follows: (i) free from neuromuscular and musculoskeletal disorders, (ii) no lower-limb serious injury including tendon or muscle tear (leading to training absence over 4 weeks) for two years prior to the study, (iii) have at least four years training and competition experience, and (iv) take part in regular resistance training for at least two years prior to the study. Participants were instructed to maintain their sleep hygiene and dietary habits, and refrain from taking stimulants and alcoholic beverages throughout the study. Testing was scheduled for the same time of the day for both experimental sessions to avoid the effects of the circadian rhythm. Furthermore, they were asked not to perform any additional resistance exercises 48-h before testing to minimize fatigue. The athletes were informed about the study’s benefits and potential risks before the experiment’s commencement and gave their written consent to participate. Moreover, they were free to withdraw from the study at any time. Participants were not told of the expected study outcomes. The study protocol was approved by the Bioethics Committee for Scientific Research at the Academy of Physical Education in Katowice, Poland (3/2021) and performed according to the ethical standards of the Declaration of Helsinki 2013. The sample size was calculated a priori using G*Power (version 3.1.9.2, Dusseldorf, Germany) and the following parameters: ANOVA, repeated measures within factors was assumed as a statistical test (one group of participants, two experimental conditions, two measurements). The statistical power was 0.8, the significance level was 0.05, and the effect size range of 0.29 to 0.39 (for interactions) based on previous studies that investigated the isometric CA and the overall PAPE effect in vertical jumping performance [1,15,18]. The power analysis indicated that a minimum sample size of between 15–26 participants was required for this study. Therefore, the 31 participants in our study would provide greater than 80% power (*p* = 0.05) to detect a small effect (small = 0.26) in jump height. 

### 2.3. Familiarization Session

**Figure 2:**
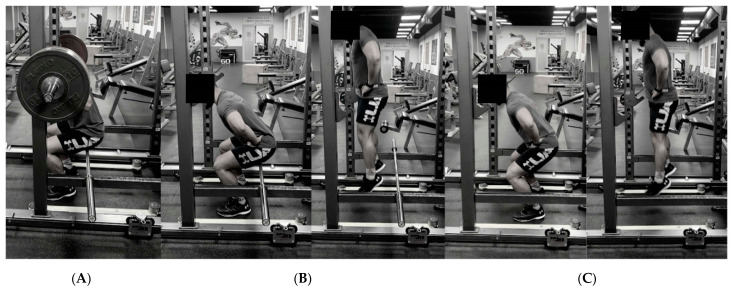
Customized exercise station to control squat depth during the conditioning activity (**A**) and the squat jump (**B**). During the countermovement jump, the depth of the squat was not restricted (**C**).

All study sessions were performed between 17:00 and 19:00. At least two days before the first experimental session, participants completed a familiarization session, including maximum isometric back squats, SJ, and CMJ. The session began with a standardized warm-up consisting of 5 min cycling and two circuits of 10 repetitions of the following bodyweight exercises: bodyweight squat, walking lunges, forward and lateral leg swings, single leg hip thrusts, and SJ. Afterward, the athletes performed 3 sets of 3 s maximum isometric back squats and 3 attempts of CMJ and SJ in randomized order. The maximum isometric back squats and SJ depth were restricted by performing them on a barbell placed on a rack that was individually adjusted and maintained at the same height during the CA and squat jump (Figure 2). During the CMJ, the depth of the squat was not restricted.

### 2.4. Experimental Sessions

After the warm-up (same as during the familiarization session), the participants performed baseline SJ and CMJ height assessments in random order. Two attempts of each jump with 30 s rest interval between them were executed. No arm swing was allowed. After 5 min of rest, the participants performed a maximum isometric back squat as a CA (EXP) or no CA (CTRL) in a randomized order. During a maximum isometric back squat, each participant was required to keep contact with the barbell on a rack behind them with individually adjusted height to ensure approximately 90°-degree knee extension (Figure 1). The maximum isometric squat was performed on an unmovable barbell loaded with a supramaximal load that did not allow any concentric movement. Positioned under the barbell, the participants were instructed to push the barbell as forcefully and as fast as possible for 3 s. Three sets of 3 maximal attempts were performed with a 3-min rest interval between sets. During the CTRL condition, the participants had to walk on a treadmill at 5 km/h for the time needed to complete the whole CA (approximately 6 min and 30 s).

### 2.5. Measurement of Squat and Countermovement Jump Height

The Optojump photoelectric cells (Microgate, Bolzano, Italy) device was used to measure jump height. It is an infrared platform with proven validity and reliability for assessing vertical jump height [24]. The device measures the jump height from flight time (9.81 × [flight time]2/8) with a frequency of 1000 Hz. The photoelectric cells were placed between the racks.

Each participant performed two CMJ and SJ without arm swing at pre-CA as a baseline and approximately in the 4th and 8th min post-CA. In the CMJ, the participant started standing with hands placed on the hips. Then, they dropped into the countermovement position to a self-selected depth, followed by a maximal effort vertical jump. The participant reset to the starting position after each jump. During the SJ, the participant had to perform a downward movement to make contact with the barbell placed behind them, maintain the tension for 3 s and afterward perform a fast-upward movement to jump as high as possible. Participants were instructed to land with both feet and knees fully extended in both the CMJ and SJ (no leg tucking was allowed) [25]. A 30 s rest interval between each attempt was allowed, and the procedure was completed after four jumps (2 of each). The jump height (based on the flight time measurement) was evaluated, and the best attempt of each jump was kept for further analysis. Due to the high inter-individual variability in the potentiation responses [26] and the individualized recovery time approach [17], the highest value obtained post-CA was also analyzed.

### 2.6. Statistical Analysis

All statistical analyses were performed using SPSS (version 25.0; SPSS, Inc., Chicago, IL, USA) and were shown as means with standard deviations (±SD) with their 95% confidence intervals (CI). Statistical significance was set at *p* < 0.05. The normality of data distribution was checked using Shapiro–Wilk tests. Relative (two-way mixed effects, absolute agreement, single rater intra-class correlation coefficient) and absolute (coefficient of variation) reliability were calculated from baseline values of both sessions. The thresholds for interpreting intra-class correlation coefficient results were: <0.5 “poor”, ≤0.5 “moderate” <0.75, ≤0.75 “good” <0.9, and ≥0.90 as “excellent” [27], whereas for the coefficient of variation, the results were: <10% “very good”, ≤10 “good” <20%, ≤21 “acceptable” <30%, and ≥30% as “not acceptable” [28]. The two-way ANOVAs (2 conditions [EXP; CTRL] × 3 time-points [pre-CA; 4th and 8th-minute post-CA; best post-CA]) were used to investigate the influence of CA on jump height. Additional two-way ANOVAs (2 × [EXP; CTRL] × 2 time-points [pre-CA; best post-CA]) were used to examine individual peak PAPE responses. In addition to the raw jump height data, the post-CA jump heights were compared to the baseline values according to the following formula: % Potentiation = Potentiated Variable (jump performed at 4th and 8th minute) ÷ Unpotentiated Variable (average baseline jumps) × 100. The percent potentiation values were used to compare the PAPE effect between CMJ and SJ. Since, in this case, the data normality was not confirmed, related-samples Friedman’s two-way ANOVA by ranks were used, and the effect size was estimated by Kendall’s coefficient of concordance. When a significant main effect or interaction was found, the post-hoc tests with Bonferroni correction were used to analyze the pairwise comparisons. Moreover, a paired-sample t-test was used to examine individual peak PAPE responses (the highest value obtained post-CA, regardless of the time). The effect sizes were determined by Cohen’s d, which was characterized as “trivial” (|d| < 0.20), “small” (0.20 ≤ |d| < 0.50), “moderate” (0.50 ≤ |d| < 0.80), or “large” (|d| ≥ 0.80) [29]. The chi-squared test was performed to identify whether there are differences between participants who obtained the best PAPE at the 4th and 8th-minute post-CA.

## 3. Results

The ICC and CV equaled, respectively, 0.84 (95%CI: 0.66 to 0.92) and 4.5% for SJ height and 0.93 (95%CI: 0.85 to 0.97) and 2.6% for CMJ height. The chi-squared test indicated that the distribution of participants who obtained their best PAPE response did not differ between the 4th and 8th-minute post-CA (x^2^(2) = 0.065; *p* = 0.799) (Table 2). 

A repeated measures two-way ANOVA determined a statistically significant interaction for SJ (F(2,60) = 3.629; *p* = 0.033; η^2^ = 0.108) and CMJ height (F(2,60) = 7.198; *p* = 0.004; η^2^ = 0.194).

**Figure 3:**
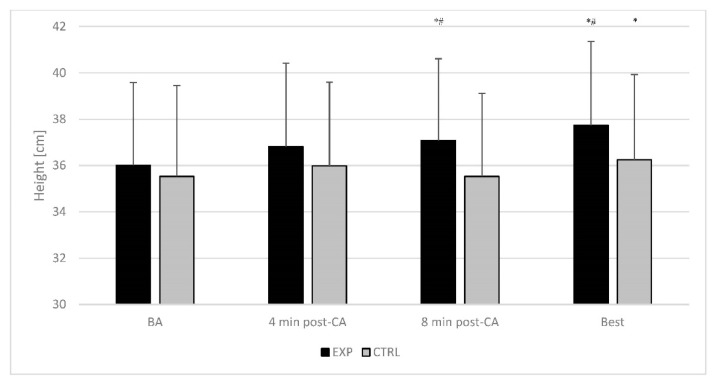
Time course of change of squat jump height. The error bars display the standard deviation. *—significant difference in comparison to baseline value within the condition; ^#^—a significant difference in comparison to corresponding time-point during the control condition. BA—baseline; CA—conditioning activity; EXP—experimental condition; CTRL—control condition; BEST—individual peak post activation performance enhancement response.

The post-hoc comparisons showed that SJ height was higher in the 8th minute (*p* = 0.004; ES = 0.31; $\bar{X}$ = 37.1 ± 3.5 cm, 95%CI: 35.8 to 38.4 cm; Δ = +3.1 ± 5.0%) than baseline ($\bar{X}\;$= 36.0 ± 3.6 cm; 95%CI: 34.7 to 37.3 cm) during the EXP condition. Furthermore, SJ height during the EXP condition was significantly higher in the 8th minute (*p* = 0.002; ES = 0.45; $\bar{X}$ = 37.1 ± 3.5 cm, 95%CI: 35.8 to 38.4 cm; Δ = +4.8 ± 7.8%) compared to the 8th minute ($\bar{X}$ = 35.5 ± 3.6 cm; 95%CI: 34.2 to 36.8 cm) in the CTRL condition (Figure 3).

**Figure 4:**
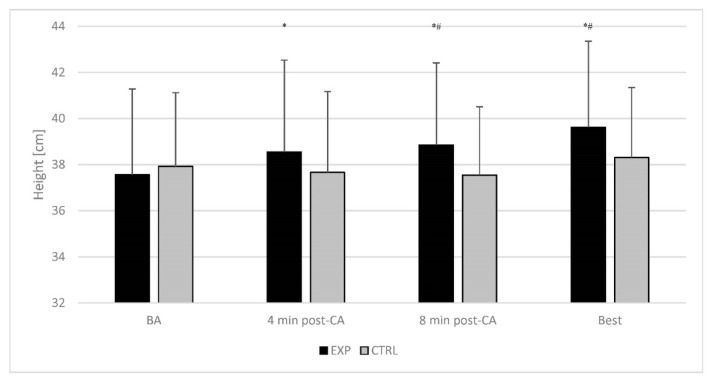
Time course of change of countermovement jump height. The error bars display the standard deviation. *—significant difference in comparison to baseline value within the condition; ^#^—a significant difference in comparison to corresponding time-point during the control condition. BA—baseline; CA—conditioning activity; EXP—experimental condition; CTRL—control condition; BEST—individual peak post activation performance enhancement response.

The post-hoc comparisons for CMJ height revealed that it was significantly higher in the 4th (*p* = 0.001; ES = 0.23; $\bar{X}$ = 38.5 ± 4.0 cm, 95%CI: 37.1 to 40.0 cm, Δ = +2.7 ± 3.7%) and 8th minute (*p* = 0.005; ES = 0.32; $\bar{X}$ = 38.8 ± 3.6 cm, 95%CI: 37.5 to 40.2 cm; Δ = +3.6 ± 5.7%) in comparison to baseline ($\bar{X}$ = 37.6 ± 3.7 cm; 95%CI: 36.2 to 38.9 cm) during the EXP condition. Moreover, the CMJ height was significantly higher at the 8th minute (*p* = 0.006; ES = 0.39; $\bar{X}$ = 38.8 ± 3.6 cm; Δ = +3.0 ± 6.3%) during the EXP condition compared to the same time-point ($\bar{X}$ = 37.9 ± 3.2 cm; 95%CI: 36.7 to 39.1 cm) during the CTRL condition (Figure 4).

### Peak Performance

A repeated measures two-way ANOVA determined a statistically significant interaction for SJ (F(1,30) = 4.251; *p* = 0.048; η^2^ = 0.124) and CMJ height (F(1,30) = 17.700; *p* < 0.001; η^2^ = 0.371) (Figure 3 and Figure 4).

The post-hoc comparisons for SJ height revealed that it was significantly higher in the best time-point compared to the baseline ($\bar{X}$ = 36.0 ± 3.6 cm; 95%CI: 34.7 to 37.3 cm) during the EXP (*p* < 0.001; ES = 0.47; $\bar{X}$ = 37.7 ± 3.6 cm; 95%CI: 36.4 to 39.1 cm; Δ = +4.9 ± 4.9%) and CTRL (*p* = 0.038; ES = 0.21; $\bar{X}$ = 35.5.0 ± 3.9 cm; 95%CI: 34.1 to 37.0 cm; Δ = +2.5 ± 5.8%) condition. Furthermore, the SJ height was significantly higher in the best time-point during EXP (*p* = 0.016; ES = 0.38; $\bar{X}$ = 37.7 ± 3.6 cm; 95%CI: 34.7 to 37.3 cm; Δ = +3.5 ± 8.4%) than in the CTRL ($\bar{X}$ = 36.3 ± 3.7 cm; 95%CI: 34.9 to 37.6 cm) condition. Moreover, the CMJ height was significantly higher at the best time-point than at the baseline (EXP: $\bar{X}$ = 37.6 ± 3.7 cm; 95%CI: 36.2 to 38.9 cm and CTRL: $\bar{X}$ = 37.9 ± 3.2 cm; 95%CI: 36.7 to 39.1 cm) during EXP (*p* < 0.001; ES = 0.53; $\bar{X}$ = 39.6 ± 3.7 cm; 95%CI: 38.2 to 41.0 cm; Δ = +5.6 ± 4.7%) and best time-point during CTRL (*p* = 0.002; ES = 0.38; $\bar{X}$ = 38.3 ± 3.0 cm; 95%CI: 37.2 to 39.4 cm; Δ = +3.1 ± 5.2%) condition.

Friedman’s test did show significant differences in percent potentiation of jump height (test = 17.361; *p* = 0.001; Kendall’s W = 0.187). Pairwise comparisons indicated a non-significant difference in percent potentiation of jump height between CMJ and SJ (*p* = 0.922) during the EXP condition. 

## 4. Discussion

The results of this study indicate that a maximum isometric squat, used as a CA, improved SJ and CMJ performance effectively and to a similar degree. The utilization of SSC in post-CA was not among the factors influencing the elicitation of the PAPE effect, nor was the similarity in terms of a range of motion and lack of utilization of SSC in the CA and subsequent task.

To the best of our knowledge, this is the first study that examined the effectiveness of isometric CA on vertical jumping with and without SSC. Examining the impact of PAPE in exercises that are merely concentric has practical implications because various sports involve such movements (e.g., ski jumping, sprint starts, swimming starts). The effects of an isometric CA on performance during exercises involving eccentric-concentric and concentric-only muscle actions would therefore be intriguing and practically significant. It has been proven that movements that are done after a prestretch provide more power output than movements that are merely concentric [30]. Increased neural drive, the storage and use of elastic energy, and contractile potentiation are all linked to improved performance in the concentric contraction preceded by an eccentric contraction and are related to the SSC [31]. Therefore, it is possible to hypothesize that the potentiating effect of a CA without using an SSC may vary depending on whether the movement involves or does not entail such a cycle. Studies so far indicate that isometric CA effectively elicits PAPE in CMJ [15,19,32,33]; far less evidence exists in the case of SJ [20]. Arabatzi et al. [20] showed enhanced SJ after maximal isometric squat only in males, with no effects in females, preadolescents, and adolescents of both sexes. These findings may suggest that the influence of strength level and sex factors might be more present in PAPE protocols that do not involve SSC. Although prior research has shown that sex has no impact on the PAPE effect [2,34,35], there might be a need for slightly different training parameters for females to optimize the PAPE effect in non-SSC tasks. The results might differ because it should be considered that in purely concentric tasks, the athlete maintains a certain period of time in muscular tension. Therefore, it cannot be ruled out that it might lead to the onset of slight fatigue, which could influence the PAPE effect. Nevertheless, the eccentric-concentric CA has been confirmed to enhance SJ performance effectively [18,36,37]. However, only Chiu et al. [18] examined both SJ and CMJ in the same group of participants. The authors showed that a back squat (eccentric-concentric) performed with 90% 1 RM improved performance for both the SSC CMJ and the SJ. This would suggest that the presence or absence of an SSC (in both CA and post-CA tasks) may not have a major impact on the level of PAPE. Nonetheless, the small number of studies yields the need for further investigations that directly compare the utilization of SSC in potentiating complexes among both sexes. 

Methodologically, one of the main principles of a correctly designed complex training protocol that effectively induces the PAPE effect is the similarity between the CA and the subsequent physical activity. Taking into account the above and the fact that the use of isometric training leads to specific adaptations in terms of strength and range of motion [38], it could be speculated that a more significant PAPE effect would be obtained in the SJ than in the CMJ in this procedure. This is mainly due to a greater similarity between the CA and the SJ in terms of a range of motion, contraction type, and a lack of stretch and shortening cycle (maintaining the squat position for 3 s before jumping, as during the CA). Therefore, although isometric contractions can lead to specific adaptations [36], they positively transfer to explosive activities. These findings are consistent with other studies and confirm the high effectiveness of isometric contractions in inducing the PAPE effect in a wide range of explosive activities [15,39]. 

The limited influence of the range of motion similarity between the CA and the following task on the magnitude of the PAPE effect is partially consistent with studies of Tsoukos et al. [40]. The mentioned study showed an increase in CMJ height (participants were instructed to bend the knee up to 90° degrees) after a maximum isometric squat at 140° knee extension but not after a 90° extension. However, contrary to the study by Tsoukos et al. [40], the maximum isometric squat at 90° of knee extension effectively improved both vertical jumps. This might be related to the different rest intervals used during the CA in those study protocols. In the study by Tsoukos et al. [40], a 1-min rest interval between CA sets was used, whereas it was 3 min in the current study. Tsoukos and colleagues [40] also assessed the effect of the applied knee flexion on the level of fatigue by the reduction in force during a 15 maximum isometric squat (in a separate session). They showed that the decrease in force was greater during the 90° than 140° knee extension. Considering that the level of CA-induced fatigue has a crucial impact on the PAPE effect [1,41], this may explain why the CA used in this study contributed to the improvement of vertical jump height, yet was ineffective in the study by Tsoukos et al. [40]. Therefore, it seems that exceeding a specific range of motion (particularly exercising at longer muscle length), even if it reflects the subsequent task, will be unfavorable due to excess fatigue, limiting potentiation, and the PAPE effect.

A finding that is also worth mentioning is that the highest PAPE response was reported when individual optimal rest intervals were analyzed. This study confirms high inter-individual variability in the potentiation responses [6,26,42] because, in half of the participants, the optimal rest interval was reported at the 4th minute, whereas in the other half it was at the 8th-minute post CA. Therefore, this justifies the need for an individualized assessment of the recovery time approach [37]. Nevertheless, the determination of individual potentiation responses can be time-consuming, especially in team sports, and disrupt the structure of the training process. Therefore, referring to the results of our study, in such a situation, a rest between 4 to 8-min following the CA might be suitable to reap benefits from the PAPE effect. 

In addition to its strengths, like a large group of participants compared to previous research [6,12,14,43], the present study has several limitations which need to be addressed. The main limitation is that we did not measure the knee flexion during the CMJ; therefore, we cannot be sure that it was significantly different from that obtained during a squat jump. Nevertheless, it was shown that the preferred depth during the CMJ is shallower than 90° [44]. In addition, since no physiological assessments were made, we cannot indicate which mechanisms contributed to the observed performance improvement. Moreover, we only assessed the height of the jump. Including kinematic variables could provide additional data on the possible PAPE effect regarding the velocity or power output. Finally, the results of this study refer to semi-professional male athletes; therefore, the extrapolation of the current findings to other populations should be performed with caution. Taking the above into account, the impact of gender, training experience, strength level, and utilization of SSC in both CA and post-CA tasks warrant further, more detailed investigations.

## 5. Conclusions

The results of this study indicate that maximum isometric squats can be effectively used for the acute improvement of both the SJ and CMJ performance among semi-professional male handball and soccer players. This finding suggests that the presence or absence of an SSC in both CA and post-CA tasks does not significantly impact the level of PAPE.

## Figures and Tables

**Table 1:** Descriptive characteristics of the study participants.

Age [years]	19 ± 2
Body mass [kg]	76.6 ± 11.5
Body height [cm]	179 ± 5
Resistance training experience [years]	4 ± 2

**Table 2:** The participants’ distribution at which time-point they obtained the best PAPE response on applied conditioning activity.

Post-CA	Time Point	
	4 min	8 min
CMJ [n]	16 (52%)	15 (48%)
SJ [n]	17 (55%)	14 (45%)

CA—conditioning activity; CMJ—countermovement jump; SJ—squat jump.

## Data Availability

The datasets used and analyzed during the current study are available from the corresponding author upon reasonable request.
